# FIB-FESEM and EMPA results on *Antoninianus* silver coins for manufacturing and corrosion processes

**DOI:** 10.1038/s41598-018-28990-x

**Published:** 2018-07-16

**Authors:** María Teresa Doménech-Carbó, Francesca Di Turo, Noemí Montoya, Fiorenzo Catalli, Antonio Doménech-Carbó, Caterina De Vito

**Affiliations:** 1 Institut de Restauraciò del Patrimoni, Universitat Politécnica de Valéncia, Camì de Vera 14, 46022 Valéncia, Spain; 2 Department of Earth Sciences, Sapienza University of Rome, P.le Aldo Moro 5, Rome, Italy; 3 Departament de Quìmica Analìtica, Universitat de Valéncia, Dr. Moliner, 50, 46100 Burjassot (Valéncia), Spain; 4 Via Attilio Friggeri 95, 00136 Rome, Italy

## Abstract

A set of ancient *Antoninianus* silver coins, dating back between 249 and 274 A.D. and minted in Rome, Galliae, Orient and Ticinum, have been characterized. We use, for the first time, a combination of nano-invasive (focused ion beam-field emission scanning electron microscopy-X-ray microanalysis (FIB-FESEM-EDX), voltammetry of microparticles (VIMP)) and destructive techniques (scanning electron microscopy (SEM-EDX) and electron microprobe analysis (EMPA)) along with non-invasive, *i*.*e*., micro-Raman spectroscopy. The results revealed that, contrary to the extended belief, a complex Ag-Cu-Pb-Sn alloy was used. The use of alloys was common in the flourishing years of the Roman Empire. In the prosperous periods, Romans produced Ag-Cu alloys with relatively high silver content for the manufacture of both the external layers and inner nucleus of coins. This study also revealed that, although surface silvering processes were applied in different periods of crisis under the reign of Antoninii, even during crisis, Romans produced Antoninianus of high quality. Moreover, a first attempt to improve the silvering procedure using Hg-Ag amalgam has been identified.

## Introduction

Studies about coinage have proved to be a valuable tool for a better understanding of the technological aspects, helping archaeologists for numismatic analysis^1,2^ as well as useful for conservation and restoration of metal artefacts^3–5^, aiming to preserve these objects from further damage.

In ancient time monetary system was, in general, a challenging task, influenced by economic crisis, availability of metal sources and monetary reforms^6,7^. In particular, the Ag content of the currency minted for about sixty years by the Antoninii emperors reflected the economic health or crisis of the Roman Empire.

Under the reign of Caracalla (*Marcus Aurelius Antoninus*), *ca*. 214–215 A.D., was instituted a monetary reform, which introduced a new denomination called *Antoninianus*^8^. The first imperial silver coinage was of a great production, with high content of Ag. Then, a severe debasement of mint and political affairs lead to productions of coins similar to argentiferous bronze coins until the beginning of the reign of Diocletian, who in 294 A.D. reintroduced an high quality of silver coinage^9^. During the production of *Antoninianus* (from 214 to 274 A.D.), Rome lived one of the most complexes period involving politic, economic and coinage fields.

At the beginning, the *Antoninianus* denomination was a silver-rich coin (up to 80% of Ag), but gradually was devalued becoming a bronze coin with a very low content of silver (about 2–3% of Ag). Historians referred^10^ that Caracalla minted this denomination with a medium content of Ag around 50%. Lately, this content decreased progressively, reaching the lowest values (about or less than 5%) under the reigns of *Gallienus* and *Aurelianus* (270–275 A.D.) as consequence of a economic crisis^9^.

Literature data report that the decreasing of Ag content is followed by an increasing of Cu, Pb and Sn content^11–13^. The latest production of *Antoninianus* was characterized by specimens of irregular weight and low-quality of manufacturing. However, the impression of a good values denomination had to be ensured, so the practice to cover with layers of Ag-enriched copper alloy on the coin surface was common^8,11–13^. The silvering of the coin surface, made in several ways^14^, was then necessary to perform due to the difficulties regarding the supply of raw materials and the needs to save Ag. This process guaranteed at the coins a look similar to pure silver and it was the unique possible solution to produce large amounts of coins for the entire territories of the Empire during periods of crisis. Therefore, the silvering process represented an important technological level for Roman coinage as it suggests a high economic value and a visual perfection of the coins^15^. Nevertheless, there is still a broad debate about when silvering process began a common practice.

Numerous methods were used to obtain a good silver surface, *e*.*g*., a mechanical attachment of the foil by hammering, tin soldering, lead or Cu-Ag eutectic (low melting point metals) and Hg-Ag amalgams^14^. This latter method was of a particular interest for the researchers and object of an exhaustive research of Anheuser *et al*.^15–17^. In the latest years of Roman Empire, the Hg-Ag amalgam was occasionally tested^8^. However, it is not clear if the amalgam was used in Europe before the Middle Age^6,9^. Probably this process was tried during the minting of the *Antoninianus* denomination, but there is still persists a doubt as there are not certain evidences. Indeed, the Hg-Ag amalgam method will become a common process of silvering in the centuries that follow the period of minting of the *Antoninianus*^18^.

Despite these historical relentless, in our knowledge a very limited number of researches on *Antoninianus* were published, reporting results on surface analysis of few samples^19–21^. As before reported in the literature^21^, the study of such material with only surface analysis could lead to erroneous conclusions as the coins had undergone to a surface modification due to corrosion processes favoured by severe environmental conditions at which coins were subjected^22^. Furthermore, these processes cause significant differences in composition from surface to inner metal, thus giving incorrect information on the nature of the alloy.

In recent years the main challenges in studies concerning valuable samples was based on bringing out new information with non-invasive^23^, or at least, micro-destructive analysis^24–26^. Surely, it is important to preserve the matter of the object as well as the aesthetic side of the artefact according to the complex philosophy of restoration that find in Cesare Brandi his founder^27^. Therefore, non-invasive and non-destructive analyses have become almost the exclusive techniques applied in Cultural Heritage fields^28–33^. It is clear that surface analyses cannot give proper or complete answers in some critic questions as technologies of manufacturing and post-manufacturing processes, raw materials and provenance^34^. In order to answer to all these questions, it seems necessary to apply a multi-analytical strategy that combines non-invasive, nano-invasive and destructive techniques.

**Figure 1:**
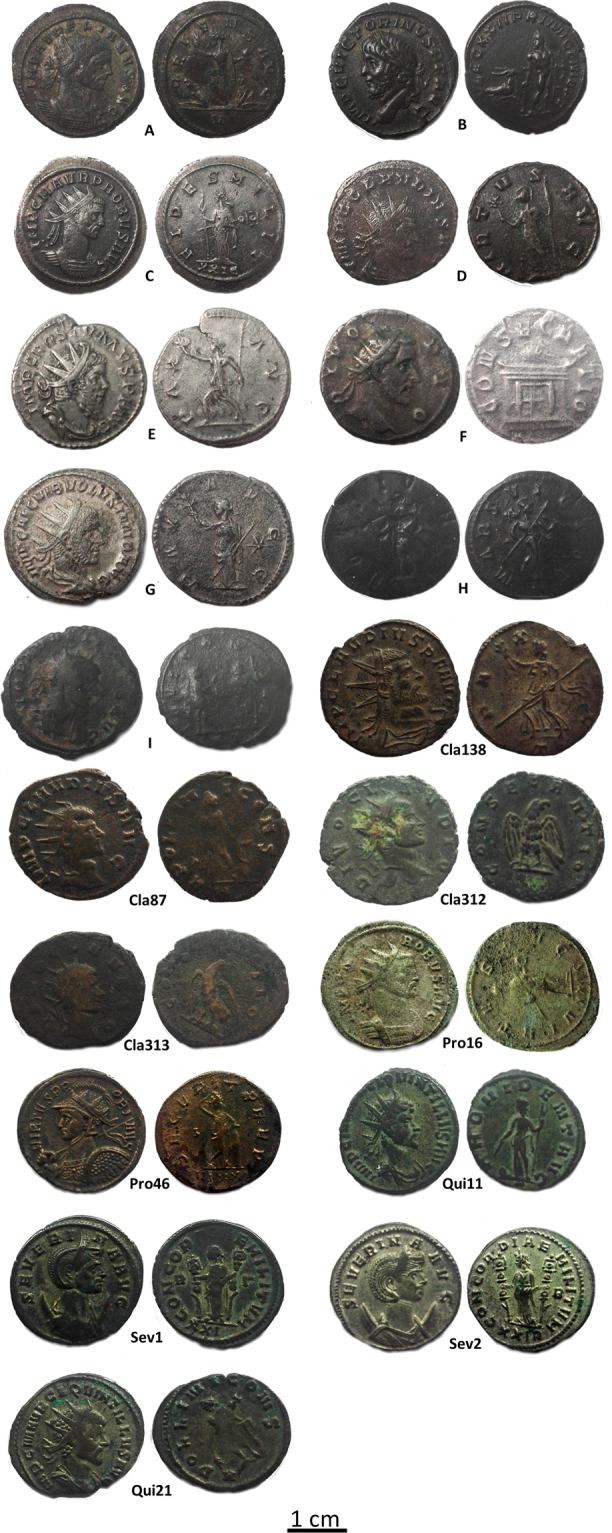
The *Antoninianus* coins studied in this work. All the historical information are reported in Supplementary Table S1.

Here we investigate a representative number of *Antoninianus* coins minted under different Roman Emperors and belonging to *Private Collections* (Fig. 1 and Supplementary Table S1). Our research aims at fill the gap of scientific knowledge regarding the ancient Ag-Cu alloy and its investigation in deep. At the same time, this research aims to answers to historical issues and to link the analytical data for numismatic questions.

The surfaces, up to 10 microns in deep, were studied with focused ion beam field emission scanning electron microscopy energy dispersive X-ray microanalysis (FIB-FESEM-EDX). The low invasiveness of FIB-FESEM technique, only a few nanograms of matter are loss, permits to analyse all coins of the *Collections*. Furthermore, such technique is very appropriate to explore the subsurface region which should be not suffered by modifications due to undocumented cleaning methods and/or preservation treatments.

Moreover, nine selected samples were analysed by Electron Micro Probe Analysis (EMPA) to acquire quantitative analysis from rim to core sections. Backscattered electron (BSE) images and X-ray maps were acquired by means of scanning electron microscopy-X-ray microanalysis (SEM-EDX), which clarified the inner microstructure of these coins.

For completeness and comparison with the inner alloy composition, the corrosion products of the surface have been characterized using micro-Raman spectroscopy and voltammetry of microparticles (VIMP).

## Results

### From rim to core investigation

To explore the distribution of Ag and Cu and to examine the microstructure of both corroded surface and inner core of the coins, a combination of SEM-EDX and EMPA analysis was employed. Although the application of EMPA technique in many fields is considered a classic method, its employment on the study of ancient materials is not common as the possibility of cutting is generally avoided. In our case to study manufacturing processes, we prepared cross-sections from rim to core of nine coins. The results obtained in the set of cross-sectioned coins enabled to distinguish two main groups on the basis of their microstructure, chemical composition and the degree of corrosion, in agreement with that observed using FIB-FESEM-EDX and complementary techniques.

**Figure 2:**
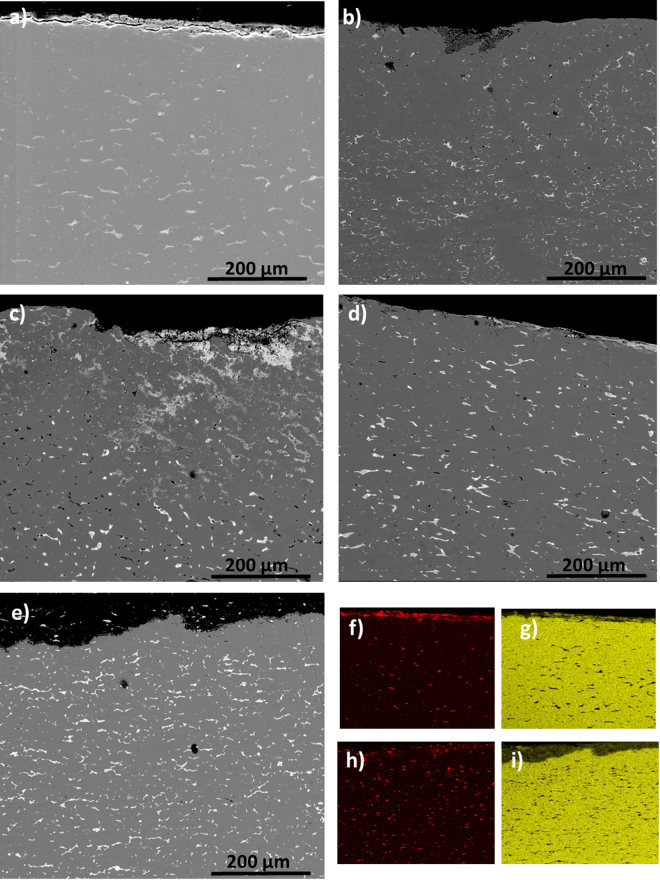
BSE images *of Antoninianus* coins (**a**) A (**b**) B (**c**) D (**d**) E (**e**) I and X-ray maps of (**f,h**) Ag and (**g,i**) Cu of coins A and I.

**Figure 3:**
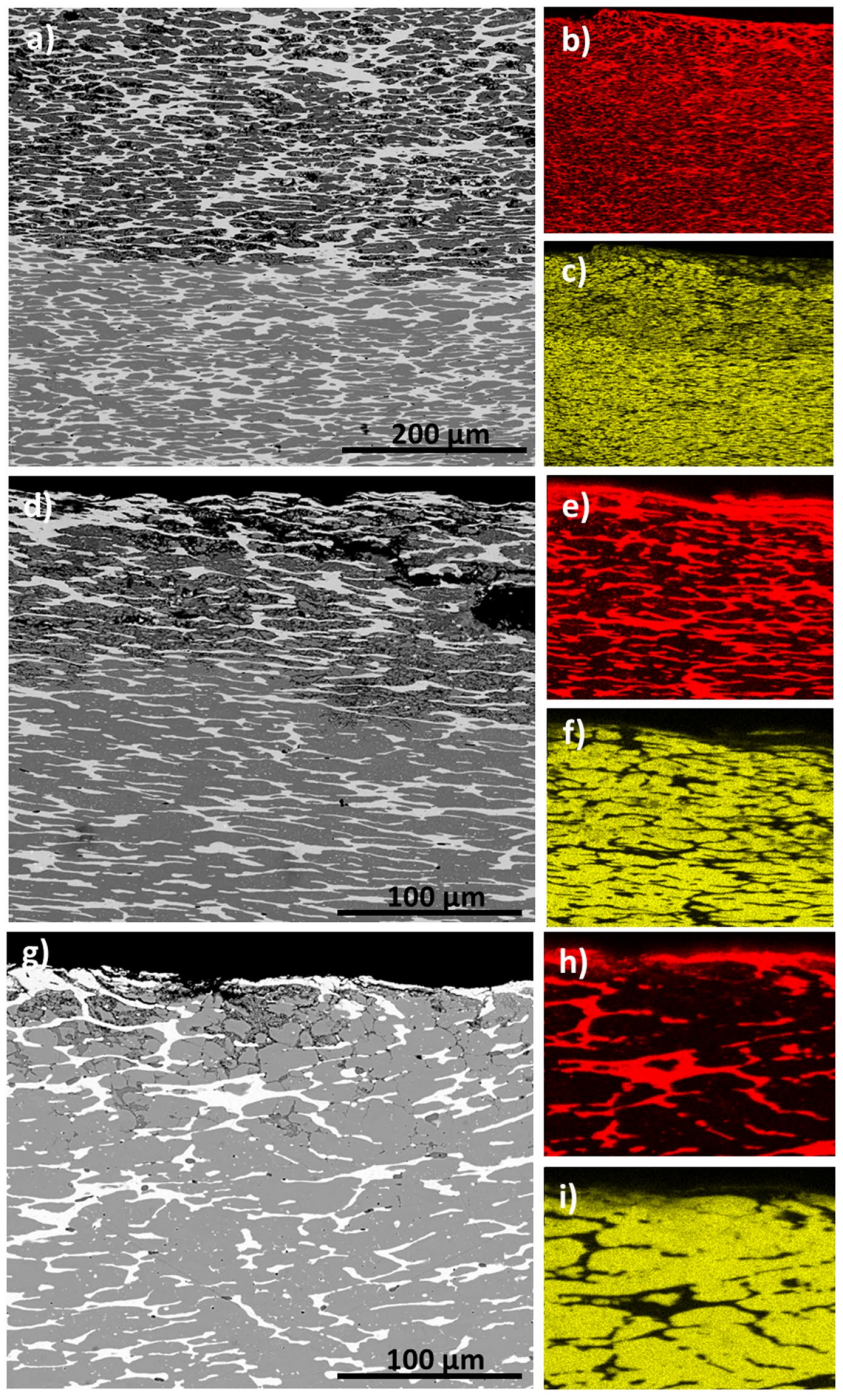
BSE images showing the microstructures of *Antoninianus* coins (**a,d,g**) C, G and H and their X-ray maps of (**b,e,h**) Ag and (**c,f,i**) Cu.

All coins studied can be assigned to the class of Ag–Cu alloys with a bulk composition consisting of a silver-rich α-phase with Ag content up to 90% (bright-grey in Figs 2 and 3) and a dark-grey copper-rich β-phase with Cu content up to 90%. Silver-rich α-phase appears as segregated inclusions, with elongated shape, parallel to the surface of the coin. A superficial Ag-enrichment is also observed in the set of coins studied. Nevertheless, a detailed examination of the surface microstructure enabled to classify the set of coins in two different groups. A first group of coins (including coins A, B, D, E) and I showed in Fig. 2 exhibits a fine corrosion layer that affects the external surface for about 15–20 µm. As illustrated in the backscattered electron (BSE) images of Fig. 2, at few micron in deep, the bulk coin shows typical Ag-Cu alloy with Cu concentration ranging from 95 to 97% and Ag content from 5 to 3% in the dark gray areas of the β-phase, whereas in those bright gray islands of α-phase the content of Ag increases up to 90% (Table 1). Selective Cu corrosion in the surface is confirmed by X-ray maps (Fig. 2g,i).

**Table 1:** Elemental composition obtained from EDS point and area analysis performed in specific layers and phases of the cross-section, 10 μm deep, provided by the FIB-FESEM trenches on the most representative *Antoninianus* silver coins.

Sample	Composition (wt%)					
		Ag	Cu	Sn	Pb	Others
Coin A	(Area α, Fig. 4c)	6.32	86.01	2.34	1.55	1.80(C), 0.43(O), 0.08(Si), 1.47(Ga)
	(Area β, Fig. 4c)	48.66	29.51	2.06	3.15	7.19(C), 4.01(O), 0.37(Si), 0.85(Cl), 4.12(Ga)
Coin B	(Area ι, Fig. 4h)	2.09	94.08			1.55(C), 1.00(O), 0.18(Fe), 1.10(Ga)
	(Area κ_1_, Fig. 4h)	76.8	19.38			1.5(C), 1.46(O), 0.86(Ga)
	(Area κ_2_, Fig. 4h)	55.55	36.93		1.48	1.80(C), 2.11(O), 2.13(Ga)
	(Area υ, Fig. 4h)	65.48	16.62			9.80(C), 4.60(O), 0.49(S), 1.87(Cl), 0.63(K), 0.53(Ca)
Coin C	(Area λ, Fig. 4b)	1.45	97.07			1.48(C)
	(Area μ, Fig. 4b)	55.27	37.86			2.99(C), 1.10(O), 0.21(S), 0.26(Cl), 2.30 (Zn)
	(Area ν, Fig. 4b)	37.54	51.15			5.25(C), 2.44(O), 1.48(S), 2.14(Cl)
	(Area ϖ, Fig. 4b)	9.22	34.88			42.11(C), 10.16(O), 0.26(Si), 0.09(P), 0.68(S), 1.55(Cl), 0.30(P), 0.57(Fe), 1.20(Ga)
Coin D	(Area ξ, Fig. 4i)	3.28	89.89	0.98	1.74	2.39(C), 1.72(O)
	(Area ο, Fig. 4i)	51.33	42.33	0.97	1.15	1.51(C), 1.85(O), 0.86(Ga)
	(Area π_1_, Fig. 4i)	5.51	78.09	1.03	2.40	8.54(C), 4.18(O), 0.11(Si), 0.14(Cl)
	(Area π_2_, Fig. 4i)	2.70	34.18	22.69	33.25	3.91(C), 3.27(O)
Coin F	(Area γ, Fig. 4a)	12.95	83.47	1.59		0.29(O), 1.69(Ga)
	(Area δ, Fig. 4a)	79.96	20.04			
	(Area ω_1_, Fig. 4a)	63.86	19.38	12.97		2.59(O), 1.20(Fe)
	(Area ω_2_, Fig. 4a)	86.95	11.59		1.46	
Coin H	(Area ρ, Fig. 4g)	5.16	93.09			0.85(O), 0.90(Ga)
	(Area ς_1_, Fig. 4g)	59.03	33.23		5.14	1.72(O), 0.89(Ga)
	(Area ς_2_, Fig. 4g)	2.75	95.07	1.91		0.27(O)
	(Area σ, Fig. 4g)	19.18	53.50		4.67	13.75(O), 1.92(Al), 3.29(Si), 0.45(Cl), 0.92(K), 0.55(Ca), 1.15(Fe)
Sev1	(Area τ, Fig. 4b)	22.17	44.38	5.42		15.53(C), 4.49(O), 0.44(S), 0.81(Cl), 0.33(K)
	(Area φ, Fig. 4b)	32.55	53.08	2.92	1.27	2.76(C), 1.42(O), 0.67(Cl), 5.35 (Hg)
	(Area υ, Fig. 4b)	1.66	94.33	1.42		1.75(C), 0.11(O), 0.73(Ga)
Qui21	(Area ε, Fig. 4d)	0.75	96.41	0.74		1.99(C), 0.11(O)
	(Area Ξ, Fig. 4d)	20.18	38.76		3.89	29.02(C), 3.34(O), 0.15(Si), 3.10(S), 1.28(Cl), 0.27(Ca)
	(Area ζ, Fig. 4d)	7.53	72.35	1.71	1.78	14.15(C), 1.89(O), 0.59(Cl)
Pro46	(Area η, Fig. 4e)	34.89	50.42	2.73	3.18	1.49(C), 3.95(O), 0.50(Si), 0.70(Cl), 0.61(Ca), 1.52(Fe)
	(Area Ѳ, Fig. 4e)	7.10	77.02	0.82	2.97	1.75(C), 3.29(O), 0.27(Al), 0.82(Si), 0.90(Cl), 1.39(Ca), 3.71(Fe)
Cla312	(Area χ, Fig. 5c)	2.40	82.42	2.50	9.29	1.89(C), 1.31(O), 0.19(Cl)
	(Area ψ, Fig. 5c)	3.16	37.42	9.40	39.65	2.94(C), 5.29(O), 0.46(Si), 0.36(P), 0.57(Ca), 0.74(Ga)
	(Area Σ, Fig. 5c)	0.59	35.10	3.40	17.33	21.65(C), 17.45(O), 0.77(Si), 0.83(P), 0.42(Cl), 0.58(K), 1.47(Ca), 0.41(Fe)
Cla87G	(Area Γ, Fig. 4f)	1.84	95.95	1.63		1.48(C)
	(Area Λ, Fig. 4f)	9.77	89.98			0.25(O)
	(Area Δ, Fig. 4f)	24.76	74.88			0.36(O)
	(Area Ω, Fig. 4f)	85.32	14.68			

The results obtained in the second group of coins, *i*.*e*., C, G, H (Fig. 3) revealed a more complex microstructure with a variable content of Cu and Ag in each micro-domains, where α- or ß- phases prevail a more intense process of selective Cu corrosion occurs. Figure 3 shows the BSE images obtained in the cross-section of these coins, in which the higher degree of corrosion of the second group of coins respect to the first one is evidenced. Here, corrosion layer achieves 200 µm in deep, resulting in the increased selective intergranular corrosion of Cu as pictured by BSE images (Fig. 3a,d,g), which, concomitantly, results in the loss of cohesion of the Cu-rich β-phase that now appears much more very fine-size grained accompanied of an increasing porosity and microfisuration. X-ray maps obtained in the outer region of these coins show a lowering of the X-ray fluorescence signal of Cu near the surface, which confirms the de-cuprification of the alloy (Fig. 3c,f,i). As consequence, silver enrichment takes place in the external surface, having concentration up to 92%. Until 200–300 μm depth (Fig. 3a,d,g), the content of Cu is lower than 90%, caused by its selective leaching, whereas the islands of the Ag noble metal remain not corroded. At greater depths than 200–300 μm, bellow the boundary between the external corroded layer and up to inner core metal, the microstructures did not present signs of corrosion. X-ray maps confirm these features and EMPA analysis shows, for some coin, content of Ag up to 90% in the α-phase islands.

### Surface analysis

**Figure 4:**
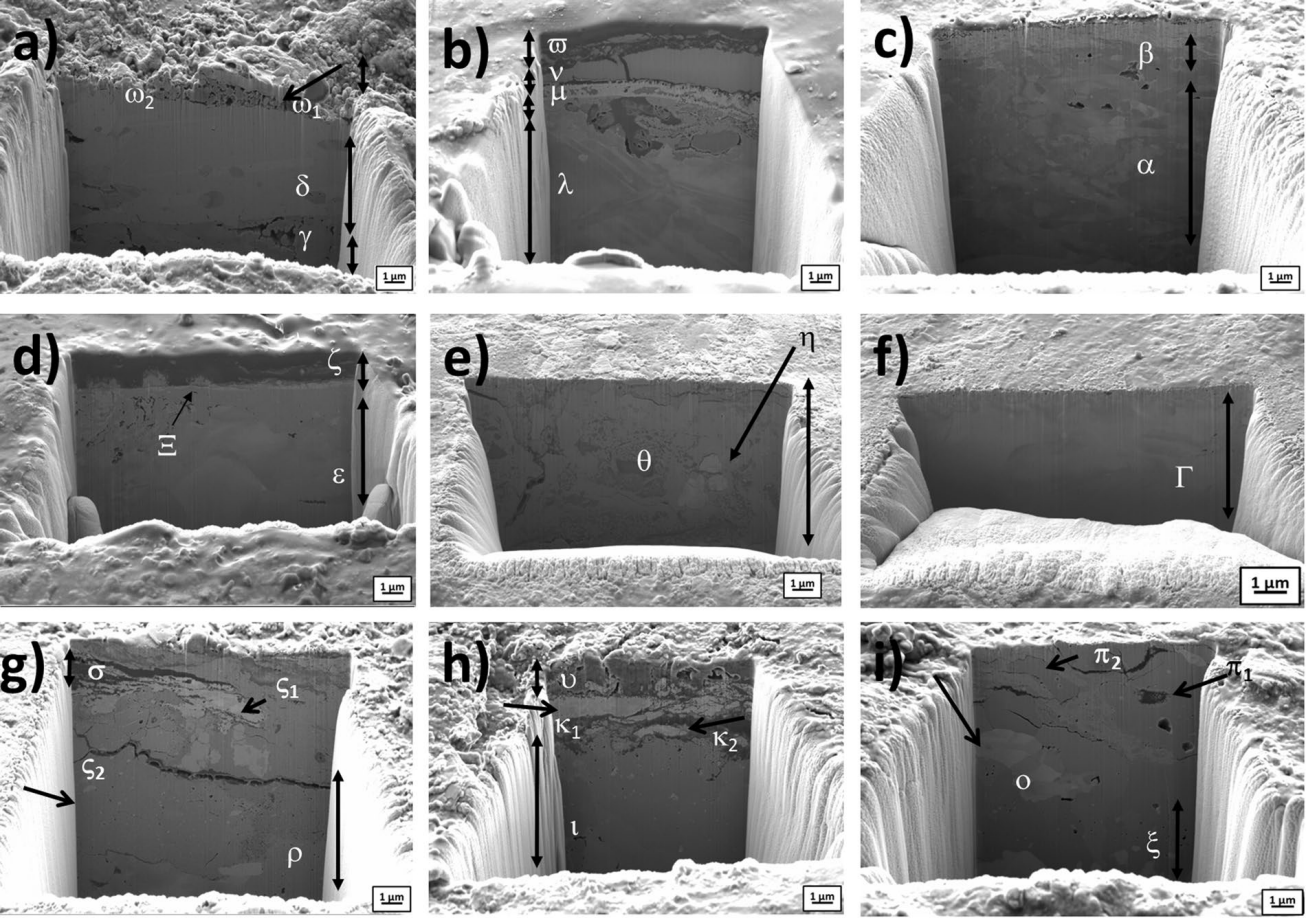
Secondary electron image of the trenches on coins: (**a**) coin F; (**b**) coin C; (**c**) coin A; (**d**) coin Qui21; (**e**) coin Pro46; (**f**) Cla87; (**g**) coin H, (image width 24 μm); (**h**) coin B, and (**i**) coin D. Points in the different layers and phases where X-ray microanalysis was carried out are labelled with Greek characters. Trench image width 15 μm.

**Figure 5:**
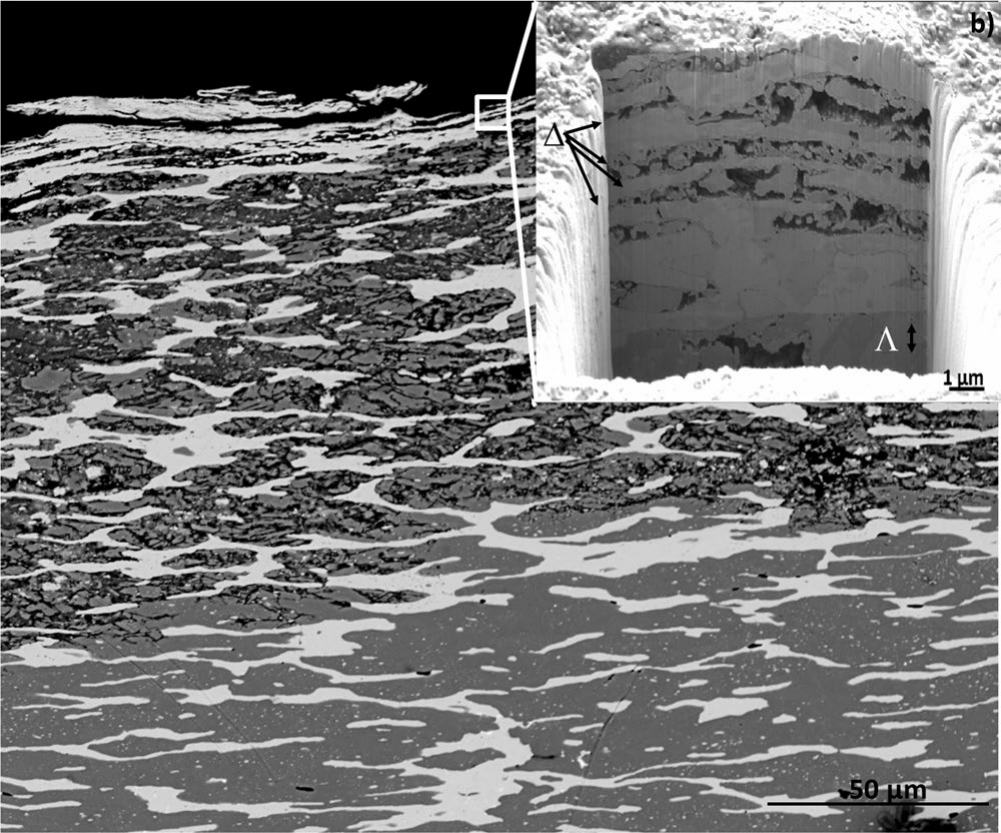
Combined image of (**a**) BSE of coin G and (**b**) a focus on a smaller area where a trench was done using FIB-FESEM. The images highlight the corrosion process and the progressive depletion of Cu as evidenced by the occurrence of secondary pores. Trench image width 18.5 μm.

FIB FESEM-EDX allows us studying the coins in nano-invasive way; therefore the analysis has been carried out on all the coins aiming to explore the complexity of microstructures and to understand the manufacture and corrosion processes affecting the outer surface (∼10 μm depth). Figure 4 shows the secondary electron images obtained in representative selected coins, which illustrate the morphological and compositional differences found in the set of coins studied. Coins F, C, A and Qui21 (Fig. 4a–d) exhibited large and elongated α-phase grains that are observed as continuous layers underneath an external more irregular corrosion layer. Coin F presented a thicker Ag-rich layered grain (area δ in Fig. 4a) of 6–4 μm thickness and composition close to that of the Ag-Cu eutectic (72 wt% Ag, 28 wt% Cu) as it can be seen in Table 1. A narrow Ag-rich layer (areas μ, β and Ξ in Fig. 4b–d, respectively), in the range 0.5–1 μm thickness, was observed in coins C, A and Qui21. In coin C, Ag-rich layer contained Cu-rich inclusions and presented a porous upper boundary. In correspondence with the presence of Cu-rich inclusions, the content of Ag is low in this grain (∼55 wt%). In all cases, the external corrosion layer consists of a mixture of corrosion products containing both exogenous (C, O, Cl, S, P, Fe, Si or Ga, the latter deposited during the formation of the trench) and endogenous (Pb, Sn, Cu, Ag) elements as result of both processes of infiltration of chemical species present in the soil environment and leaching of materials from the inner parts of the coin to the surface. The boundary between the Ag-rich layered grain and the inner core of the coin appears irregular with formation of pores and fissures and also infiltration of materials in all coins and, specially, in coin C. Interestingly, Zn was found in the segregated grains of Ag-rich phase in this coin. Underneath the Ag-rich layer, it can be seen a portion of the almost unaltered β-phase composing the bulk material of the coin with characteristic polygonal phases or grains. β-phase in the core of coin C exhibits a binary Cu-Ag composition (97.07 wt% Cu and a 1.45 wt% Ag) whereas addition of some Sn is found in the β-phase of coin F (83.47 wt% Cu, 12.95 wt% Ag and 1.59 wt% Sn), coin Qui21 (96.41 wt% Cu, 0.75 wt% Ag and 0.74 wt% Sn) or Sn and Pb in coin A (86.01 wt% Cu, 6.32 wt% Ag, 2.34 wt% Sn and 1.55 wt% Pb). In all cases, the presence of other elements such as O or C in this outer region of the bulk confirms some diffusion process of exogenous species from outside. Trench formed in coin G, that is shown in Fig. 5, presented a complex structure in the surface consisting of a package of tiny elongated grains (1–2 μm thickness) of Ag-rich α-phase alternated with Cu-rich β-phase exhibiting strong intergranular corrosion that results in the leaching of this lesser noble metal (see Table 1). The Ag content in the elongated Ag-rich grains reaches values of 85.32 wt% (Ω areas in Fig. 5). This altered morphology is in agreement with that previously described from the examination of the entire cross-section in this same coin as can be seen in Fig. 5.

A second group of coins, *i.e.* coins Pro46 and Cla87, shown in Fig. 4e,f, is characterized by almost absence of corrosion patina. Elemental composition (see Table 1) indicates that variable composition of Ag-Cu alloy was used in these coins and confirms the addition of some Sn and Pb for improving the properties of the alloy.

A third group of coins displays microstructures with abundant fractures and pores in their surface. Grains of Ag-rich α-phase, with irregular shape, can be seen dispersed in the continuous Cu-rich β-phase near the surface (see coins H, B and D in Fig. 4g–i, respectively). Pb and Sn were also detected taking part of the composition of the bulk.

**Figure 6:**
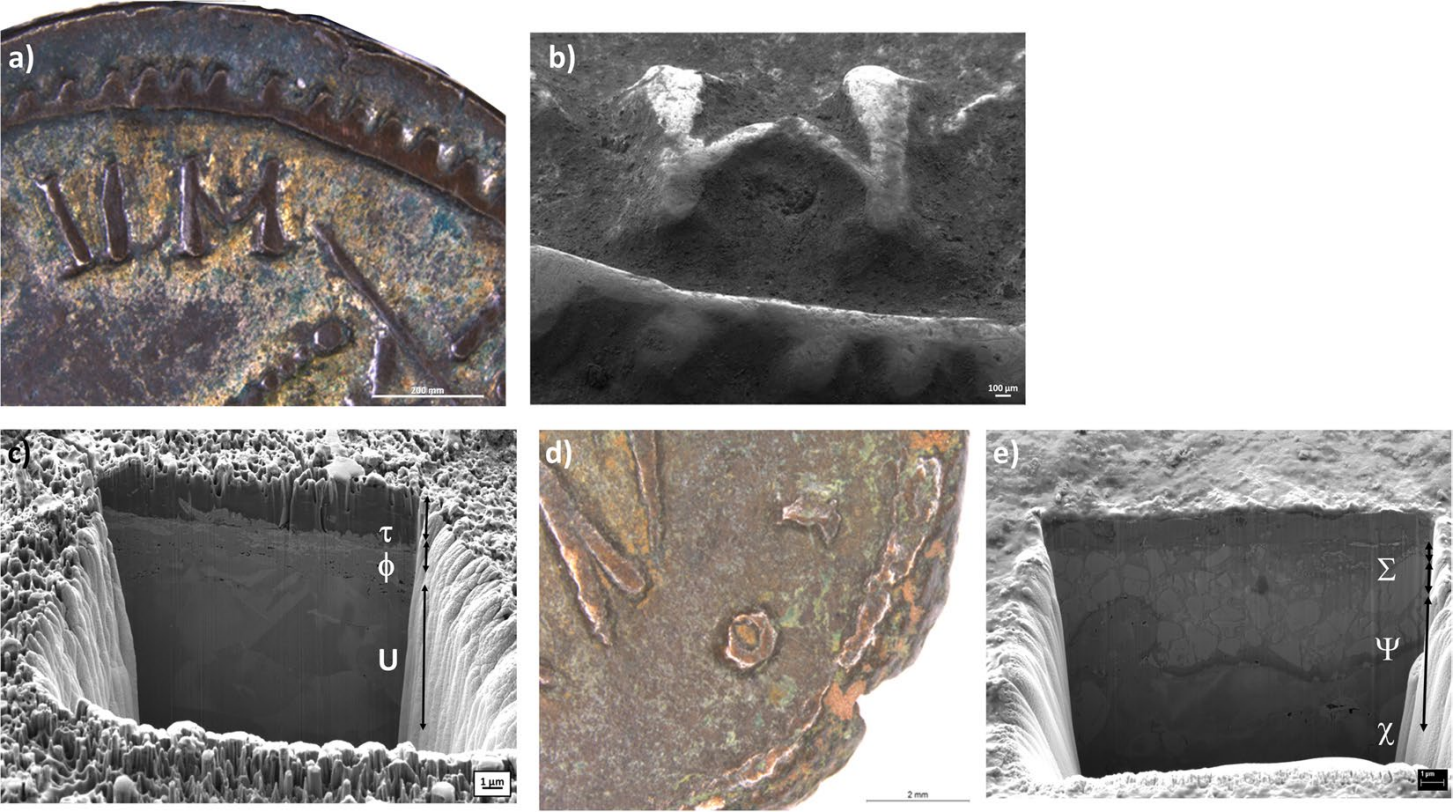
Optical microscopy (OM) photograph of Sev1 highlight golden areas on the surface (**a**). Secondary electron image of a trench performed with FIB-FESEM in the same area is shown in figure (**b**) and then the trench shows the porous morphology of Hg-Ag amalgam on the coin surface in (**c**). Optical (**d**) and secondary electron images of the trench performed with FIB-FESEM in the same area of figure (**e**) of Cla312. The trench shows the Pb square structure in the ψ analyzed area. Trench image width 18.5 μm.

Finally, two coins presented structures of particular interest. Coin Sev1 exhibited a very thin Ag-rich layer below an outer corrosion layer formed by exogenous materials (areas ϕ and υ, Fig. 6a–c) in which 5.35 wt% of Hg was found. On the other hand, coin Cla132 (Fig. 6d,e) shows a layer (∼4 μm thickness) formed by a mosaic of grains (∅ < 2 μm) enriched in Pb (39.65 wt%, area ψ in Fig. 6e).

The corrosion products of the surface of the coins were analyzed with Raman spectroscopy. Supplementary Fig. S2 compares the Raman spectra of different Antoninii coins (B, D and E, see Supplementary Table S1), taken as representative. The Supplementary Fig. S2 shows a similar surface composition for the set of coins. It can be observed in the three coins the main band of cuprite (Cu_2_O) at 218 cm^−1^ and weak and broad bands for the same compound at 400 and 630 cm^−1^. The more intense bands at 95 and 145 cm^−1^ can be attributed to silver oxide (Ag_2_O) and silver chloride (AgCl) whereas the broad bands at 430, 565, 1070 and 1100 cm^−1^ can be assigned to silver oxide (Ag_2_O) phase, in agreement with the bibliography^32^.

Supplementary Figure S3 illustrates the typical voltammetric response of sample-modified electrodes immersed into 0.25 M HAc/NaAc at pH 4.75. On scanning the potential in the negative direction, a main cathodic peak appeared at −0.10 V vs. Ag/AgCl (C_1_) which frequently looks like two or three superimposed signals. This peak was followed by ill-defined shoulders at ca. −0.40 and −0.80 V (C_2_), and a rapidly rising current ca. −1.0 V (CHER). Upon scanning the potential in the positive direction, a tall oxidation peak appeared at −0.70 V (A_2_) preceding the main anodic signal (A_1_) consisting of overlapped peaks at +0.05 and +0.20 V. This voltammetric behavior can be interpreted on the basis of recent literature on silver^1,3,35,36^, and copper^37–39^ corrosion products confirming the presence of cuprite, often accompanied by tenorite, silver oxide, silver chloride and silver sulfide as main components of the patina of coins.

## Discussion

The coins, belonging to *Private Collections*, have surfaces apparently well preserved; however, some corrosion products are present. Ag_2_O was detected using surface analysis, *i*.*e*., micro-Raman spectroscopy and VIMP, and being a stable phase, the corrosion phenomena involving the formation of non-noble *patina* could be limited.

The presence of cuprite, tenorite and lead corrosion products is in agreement with the images and compositional data provided from cross-sections and trenches analysis that point out significant content of Cu, Pb and Sn in combination with O in the outer layers of corrosion. The formation of a primary patina of cuprite followed by a secondary patina of tenorite is common in copper and bronze objects. This external layer has also been reported in replication experiments of surface silver enrichment of silver-copper alloys^40^.

Although micro-Raman spectroscopy and VIMP data are in agreement in the diagnosis of the corrosion products of copper and silver on the surface, they do not highlight substantial differences among coins. Surface studies and, in particular, those on silver coins aiming to reconstruct ancient manufacturing processes, can be misled by silvering (*see infra*) due to the surface enrichment of the precious metal^21,22,41^. On the other hand, the corrosion processes may lead to a surface composition very different from the bulk alloy. Then, surface analysis, as expected, does not allow going beyond a characterization of surface corrosion and cannot explore the core of the coins.

The analysis of cross-sections of the coins allows studying morphological and compositional variations along the entire section of the coin. As it is shown in Figs 2 and 3, the coin flan consists of a Ag-Cu alloy that underwent phase separation during cooling due to their very low solubility at room temperature. The primary phase is a copper-rich β-phase (Cu content >80 wt%). The secondary phase Ag-rich can be seen as grains dispersed in the primary phase. Their elongated shape is due to the striking of the blank during the minting process^42^. Copper was widely used in ancient time for the production of silver artifacts; despite it is a melting-point depressant diffusing easily in silver^43^.

The inner part of the flan of all coins contains lead and tin in a very low amounts, excluding the hypothesis of archaeologist who believed that also the *Antoninianus* was made of a complex Ag-Cu-Pb-Sn alloy, common in coins minted in the studied period^8^. Only Cla312 sample (Fig. 3d,e) has an high content of Pb as testified by its microstructure that contains hemihedral segregates and unalloyed copper inclusions. In this coin the content of Ag is very low also in the surface layer. The very high content of Pb suggest the voluntary addition of this metal, excluding the accidental occurrence due to the use of *argentiferous* galena for the extraction of Ag^44^. This difference is reasonable as the Cla312 was minted in memory of *Divus Claudius*, after the 270 A.D. (Supplementary Table S1) and therefore, it differs from the other *Antoninianus* coins.

The complex microstructure of the studied coins, with Ag-rich elongated grains containing Pb and Sn, and eventually Zn, (see BSE imaging and X-ray maps in Figs 2 and 3), is the results of the low solubility of these metals in Cu and *vice versa* at room temperature, which results in the segregation of α and β phases. This microstructure promoted processes of selective intergranular corrosion of copper, causing an important secondary porosity. The concomitant leaching and oxidation of Cu is mainly evident in the external areas where an outermost layer cuprite-rich is formed. This process has taken place at different extent in the set of studied coins. Thus, the five coins of the first group exhibit a low degree of corrosion. In contrast, the four coin of the second group present a greater corrosion than that of the coins of the first group at the expense of Cu (Fig. 3a–i). This behavior is tentatively associated with the increase in the Ag content in the alloy in the latter group, which should result in a more ductile alloy that favors dislocation of grains by mechanical stress during minting (as it is evidenced by the elongated shape of Ag-rich grains in BSE images) for both α and β phases, thus, promoting the selective intergranular corrosion of the weaker Cu-rich β phase. The post-production history could also influence this different behavior.

EMPA and FIB-FESEM-EDX analyses indicate that all these coins present enrichments of silver in the external layer up to 92% (Tables 2 and 3) concomitant to the depletion of copper with overall content ranging from 3 to *ca*. 30%. In particular, the Cu-rich β-phase (Fig. 2a–d and Tables 2 and 3) and the Ag-rich α-phase vary their compositions with respect to the bulk, resulting Ag- and Cu-enriched, respectively (Tables 2 and 3). This process sometimes induced erroneously numismatics to explain that as a continuous silvering of coins.

**Table 2:** Chemical analyses of representative coins of group 1, obtained using microprobe analysis on the external and deeper layers. Ex-layer: external layer; Int-gray: internal core (copper-rich β-phase); Int-island (silver-rich α-phase).

Coins	A			B			D				E			F		
	Ex- layer	Int- gray	Int- island	Ex- layer	Int- gray	Int- island	Outer Ex- layer	Inner Ex- layer	Int- gray	Int- island	Ex- layer	Int- gray	Int- island	Ex- layer	Int- gray	Int- island
Ag wt.%	88.01	3.30	73.20	91.88	4.47	68.50	87.61	3.24	7.98	87.36	81.60	2.62	70.30	69.60	2.75	77.60
Cu	10.88	94.50	26.03	7.06	94.73	31.20	8.00	93.57	89.06	9.70	15.60	95.51	27.01	28.88	96.83	19.60
Au	0.02	0.15	0.02	0.00	0.00	0.01	0.16	0.01	0.18	0.03	0.00	0.00	0.03	0.00	0.109	0.00
As	0.01	0.06	0.01	0.01	0.09	0.01	0.00	0.09	0.27	0.02	0.21	0.12	0.02	0.11	0.11	0.33
Pb	0.03	0.09	0.03	0.03	0.00	0.10	2.47	0.54	0.16	1.25	0.03	0.04	1.25	0.04	0.00	0.05
Sn	0.98	2.17	1.03	0.16	0.11	0.05	1.06	2.29	1.45	1.03	1.60	1.53	1.03	0.56	0.44	1.55
Ni	0.01	0.02	0.01	0.04	0.01	0.00	0.04	0.09	0.13	0.08	0.00	0.00	0.08	0.01	0.06	0.00
Zn	0.01	0.04	0.02	0.01	0.00	0.00	0.15	0.00	0.17	0.03	0.00	0.00	0.03	0.00	0.00	0.00
Fe	0.01	0.05	0.01	0.45	0.27	0.20	0.02	0.07	0.04	0.27	0.77	0.03	0.27	0.57	0.01	0.77
tot	99.96	100.37	100.35	99.64	99.68	100.07	99.52	99.90	99.43	99.77	99.81	99.85	100.02	99.77	100.32	99.90

**Table 3:** Chemical analyses of representative coins of group 2, obtained using microprobe analysis on the external and deeper layers. Ex-layer: external layer; Int-gray: internal core (copper-rich β-phase); Int-island (silver-rich α-phase).

Coins	C				G			H				
	Ex- layer	Ex- layer	Int- gray	Int- island	Ex- layer	Int- gray	Int- island	Ex- layer	Ex- layer	Int- gray	Int- gray	Int- island
Ag wt.%	90.21	14.43	9.92	91.16	88.30	2.22	91.77	91.75	87.99	3.17	1.92	79.99
Cu	5.30	81.41	88.00	5.74	6.30	98.00	4.56	4.55	8.40	93.91	0.00	17.01
Au	0.15	0.34	0.18	0.00	0.15	0.01	0.00	0.00	0.00	0.00	0.00	0.00
As	0.00	0.51	0.27	0.01	0.00	0.02	0.00	0.05	0.01	0.06	0.05	0.07
Pb	2.38	0.18	0.16	1.75	2.17	0.17	1.38	2.38	1.19	0.12	0.04	1.03
Sn	1.71	3.03	1.45	1.48	1.55	0.00	2.35	1.75	2.39	2.05	1.12	2.71
Ni	0.04	0.00	0.13	0.09	0.04	0.07	0.03	0.00	0.00	0.00	0.00	0.00
Zn	0.15	0.08	0.17	0.05	0.27	0.02	0.03	0.04	0.06	0.09	0.04	0.00
Fe	0.00	0.09	0.04	0.03	0.68	0.04	0.00	0.02	0.00	0.04	96.70	0.00
tot	99.94	100.05	100.32	100.32	99.46	100.55	100.13	100.54	100.03	99.44	99.88	100.81

The quantitative EMPA and FIB-FESEM-EDX analyses support the hypothesis that Romans used the Cu-Ag alloys for the production of both the external layers and inner blanks not only during flourishing years but also during crisis periods contrarily to the traditional idea that they used Cu-Ag alloys during decadence periods to produce, exclusively, the external layers for lowering production costs. It is possible to suppose that the silvering was actually used to enhance the aspect of silver coins but in some cases the percentage of Ag into the alloy still remains high despite the historical sources^15^. These coins were minted in the late period of the Antoniniani’ Emperors and the literature about that age, which reported the manufacture of the coins, was of poor quality^8^, suggesting a very low content of Ag in the blank. In this research we have demonstrated that the Romans, also during periods of crisis, produced *Antoninianus* of high quality.

It is of worth to note that Sev1 coin differs from the others for its gilded aspect at macroscopic scale (Fig. 6a,b). The FIB-FESEM-EDX data reveal the presence of Hg on the surface of this coin, confirming the use of Hg-Ag amalgam for silvering (Fig. 6c). The occurrence of Hg supports the hypothesis that in the studied period the silvering process was carried out by amalgamation. During the economic crisis of the Roman Empire to save metals meant a support for solders and defenses, thus this led to reduce the content of Ag into the silver coins, specially, during the reign of *Gallienus*^8^. However, the aspect of silver coins had to be similar to that of high quality and a surface enrichment in Ag was strictly necessary. Silvering is a very open question as it is not still clear when it became a common practice. In ancient times, there were available several processes for plating silver coins such as mechanical attachment of the foil by hammering, soldering by using tin, lead or Cu-Ag eutectic (low melting point metals) and Hg-Ag amalgam^17,18^. At first, it was presumed that amalgam silvering wasn’t adopted in Europe before the Medieval Age^8,9^ but actually this technique was used by Romans even if there are a very few evidences in Roman coinage^8,13^ and in particular for *Antoninianus* denomination. The presence of Hg on the surface of Sev1 can be the result of uncontrolled heating process usually used to remove Hg through boiling (357 °C)^9,14,45^. We can consider this as a first attempt of silvering employing the amalgam. The considered methodology of silvering will become more usual in the subsequent centuries^18^ and it is not easy to find evidences of the use of amalgam in the late *Antoninianus*, when the first attempt to have a better silver-plating was done^8^.

This research opens a new scenario in which multi-technique approaches based on the combination of surface analysis techniques such as FIB-FESEM-EDX, micro-Raman spectroscopy or VIMP and cross-sectional analysis by means of EMPA and SEM-EDX are used to characterize not only the composition of the alloy and complex microstructures but also to complement archaeometric analysis and historical issues.

## Methods

- FIB-FESEM Zeiss (Orsay Physics Kleindiek Oxford Instruments) model Auriga compact equipment enables to analyze the patina of the all 19 coins. The operating conditions used: voltage, 30 kV and current intensity, 500 µA and 20 nA in the FIB for generating the focused beam of Ga ions. The Ga beam impacts perpendicularly to the plane of the vertical wall of the trench by tilting 54° the stage where is placed the coin. Were used a voltage of 3 kV for photographs with FESEM. Electron beam was optimally focused for acquiring images, which were automatically corrected by the software that performs “tilt compensation”. X-ray microanalysis was performed in the trenches operating with an Oxford-X Max X-ray microanalysis system coupled to the FESEM controlled by Aztec software. A voltage of 20 kV and a working distance of 6–7 mm were applied. Semi-quantitative microanalysis was carried out by the ZAF method to correct inter-elemental effects. The counting time was 100 s.
- SEM investigation was done using a FEI-Quanta 400 (SEM-EDS) instrument, operating at 20 kV, equipped with X-ray energy-dispersive spectroscopy (Department of Earth Sciences, Sapienza University of Rome, Italy). SEM imaging and X-ray maps were performed to evaluate microstructure, corrosion layer and elemental distribution from rim to core.
- EMP analysis was done using a Cameca SX50 electron microprobe equipped with five wavelength-dispersive spectrometers (CNR–IGAG, Rome, c/o Department of Earth Sciences, Sapienza University of Rome). The operating conditions were: accelerating voltage 15 kV, beam current 15 nA and beam size 10 µm on matrix and glaze, and 2–5 µm on inclusions. Element peaks and background were measured with counting times of 20 and 10 s respectively. Chalcopyrite was used as a reference standard for Cu (LIF), synthetic Au20 for Ag (PET), galena for Pb (PET), cassiterite for Sn (PET) and Matrix corrections were calculated by the PAPmethod^46^, with software supplied by Microbeams Services. The analytical error was ∼1% rel. for the major elements, and it increases as their concentration decreases. The detection limits under the specified working condition range between 0.01 and 0.1 wt%.
- Raman spectra of different coins were obtained by means of a XPlora Horiba MTB model and a 532 nm laser as excitation with maximum power of 90 mW. The samples were measured in back-scattering geometry at room temperature. A 100 confocal microscope objective was used to focus the excitation laser on the sample and collect the scattered light to the spectrometer. More than 3 different areas were analyzed per sample, to obtain representative results. Exposure time, number of acquisitions and laser power varied among 5–20 s, 10–50 and 30–80 mW, respectively. Data acquisition was carried out with the LabSpec 6 Spectroscopy Suite from Horiba MTB.
- Electrochemical experiments were performed at 298 K in a three-electrode cell using a CH I660C device (Cambria Scientific, Llwynhendy, Llanelli UK) using air-saturated aqueous 0.25 M sodium acetate buffer (Panreac) at pH 4.75 as a supporting electrolyte. A platinum wire counter electrode and an Ag/AgCl (3 M NaCl) reference electrode completed the three-electrode cell. Sample-modified electrodes were prepared by pressing the graphite bar (Faber Castell HB, diameter 2 mm) onto one plane region of the surface of the coins during 5 s and then being dipped into the electrochemical cell so that only the lower end of the electrode was in contact with the electrolyte solution, as already described^1–3^. Square wave voltammetry was used as a detection mode.

## Electronic supplementary material

Supplementary Information
