# Supplementary material for: FIB-FESEM and EMPA results on Antoninianus silver coins for manufacturing and corrosion processes
Source: Sci Rep. 2018 Jul 16;8:10676. doi: 10.1038/s41598-018-28990-x (PMC6048157; doi:10.1038/s41598-018-28990-x)
Supplement: Supplementary file 1 — Supplementary Information [file 41598_2018_28990_MOESM1_ESM.pdf]

FIB-FESEM and EMPA results on *Antoninianus* silver coins for manufacturing and corrosion processes

María Teresa Doménech-Carbó, Francesca Di Turo, Noemí Montoya, Fiorenzo Catalli, Antonio Doménech-Carbó, Caterina De Vito.

**Sample**	**Emperor**	**Age (A.D.)**	**Mint**
A	*Lucius Domitius Aurelianus*	270 - 275	Rome
B	*Marcus Piavonius Victorinus*	269 - 271	Galliae
C	*Marcus Aurelius Probus*	276 - 282	Rome
D	*Marcus Aurelius Valerius Claudius*	268 - 270	Rome
E	*Marcus Cassianius Latinius Postumus*	260 - 269	Galliae
F	*Gaius Messius Quintus Traianus Decius*	249 - 251	Rome
G	*Gaius Vibius Afinius Gallius Veldumnianus Volusianus*	251 - 253	Galliae
H	*Lucius Domitius Aurelianus (?)R/ the same type incuse*	270 - 275	Rome
I	*Lucius Domitius Aurelianus*	270 - 275	Rome
Sev1	*Severina Augusta wife of Aurelianus*	270 - 275	Rome
Sev2	*Severina Augusta wife of Aurelianus*	270 - 275	Rome
Pro16	*Marcus Aurelius Probus*	276 - 282	Rome
Pro46	*Marcus Aurelius Probus*	276 - 282	Rome
Qui21	*Gaius Fulvius Quietus, son of Macrianus*	260 - 261	East
Qui11	*Gaius Fulvius Quietus son of Macrianus*	260 - 261	East
Cla138	*Marcus Aurelius Valerius Claudius*	268 - 270	Ticinum
Cla87	*Marcus Aurelius Valerius Claudius*	268 - 270	Rome
Cla312	*Divus Claudio*	After 270	Rome
Cla313	*Divus Claudio*	After 270	Rome

Supplementary Table S1 . Name of samples with their emperors, age of coinage and mint.

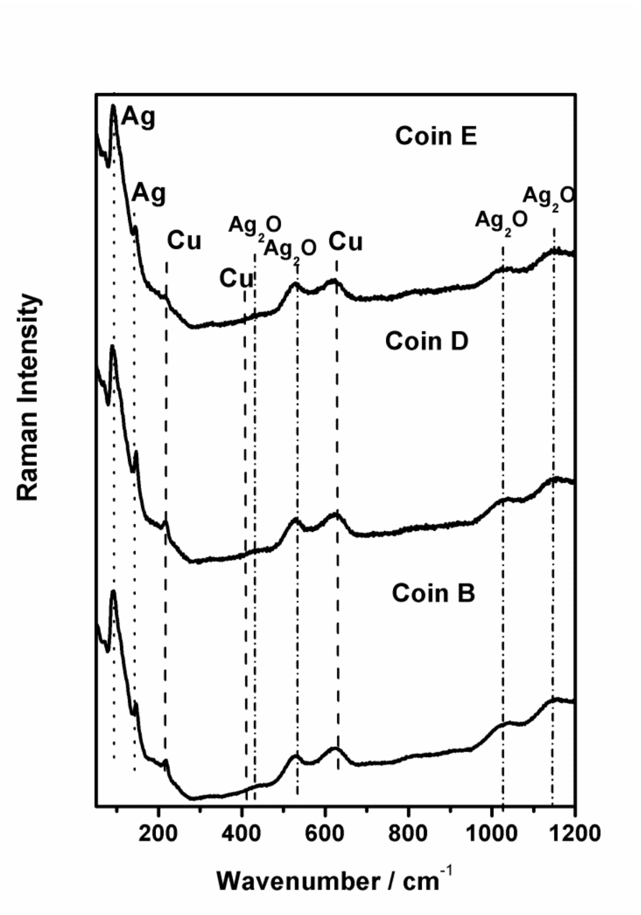

Supplementary Figure S2. Raman spectra of three coins B, D and E. It highlights the main corrosion products for all coins. (Cu is cuprite (Cu_2O), Ag is silver (I) oxide (Ag_2O) and silver (I) chloride (AgCl) and Ag_2O is silver (I) oxide phase).

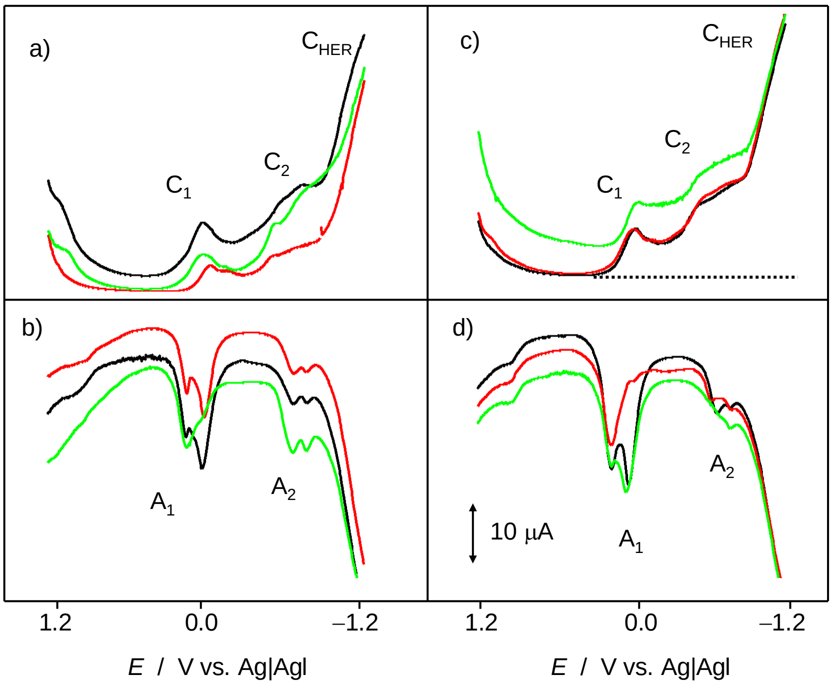

Supplementary Figure S3. VIMP analysis carried out on the patina of Ag-Cu coins. Square wave voltammograms (three independent replicate experiments) of samples from coins **a,b**) Aurelianus (A) and **c,d**) Vittorinus attached to graphite electrodes immersed into air-saturated 0.25 M HAc/NaAc at pH 4.75. Potential scan initiated at a,c) +1.25 V in the negative direction; **b,d)**- 1.25 V in the positive direction; potential step increment 4 mV; square wave amplitude 25 mV; frequency 5 Hz. Dotted lines represent the base lines for peak current measurements.
